# Supplementary material for: IDNet: An inception-like deformable non-local network for projection compensation over non-flat textured surfaces
Source: PLoS One. 2025 May 20;20(5):e0318812. doi: 10.1371/journal.pone.0318812 (PMC12091798; doi:10.1371/journal.pone.0318812)
Supplement: Attachment — Submitted filename: Response to Reviewers.pdf [file pone.0318812.s003.pdf]

Suggestion 1:
Introduction section is too short and written just like an abstract. It does not satisfy the need and requirements of an interested reader. Therefore author is suggested to rewrite the introduction section which should depicts the introduction of the field, existing methodologies and their associated issues, how the research gap is identified, your proposed approach adopts which unique technique which improves the it performance and make it novel and the contribution of their proposed approach. Author should use bullet 1 for the introduction section and so on for subsequent sections.

Response:
Thank you for your valuable feedback.
We have revised the Introduction section in accordance with your suggestions. The updated version now provides a more comprehensive overview of the field, outlines existing methodologies along with their associated challenges, and highlights the identified research gap. Additionally, we have clearly explained the unique techniques adopted in our proposed approach, how they contribute to performance improvement, and what makes our approach novel. We believe these revisions better address the interests of readers and fulfill the requirements for a robust introduction. We have also organized the content using bullet points for clarity, as per your recommendation.

Suggestion 2:
The bullet of the section Related work should be 2 and there some be some line of text between the main and subsequent heading. This suggestion should be followed in whole paper. Use the reference of the latest approaches of 2023-24 and then address the novelty of the proposed approach.

Response:
Thank you for your helpful suggestion.
We have reviewed the latest paper from 2023-2024 and incorporated relevant approaches, along with their associated techniques and limitations, into the 'Related work' section. The section now reflects the most recent advancements in the field, and we have addressed how our proposed approach overcomes the challenges highlighted in these works, thereby emphasizing its novelty. Additionally, we have followed your formatting recommendation by adding appropriate spacing between the main and subsequent headings, ensuring consistency throughout the paper.

Suggestion 3:
Figure 1,2,3,4 and 5 are missing within the manuscript.

Response:
Thank you for pointing out the issue regarding the missing figures. According to the submission guidelines provided by PLOS One (https://journals.plos.org/plosone/s/submission-guidelines#loc-figures-and-tables), figures should be uploaded as separate files rather than being included within the manuscript itself. In line with these guidelines, Figures 1, 2, 3, 4, and 5 have been uploaded as individual files to the submission system. We hope this resolves the issue, and we appreciate your understanding.

Suggestion 4:
Use a consistent formatting style for tables within the paper.

Response:
Thank you for your valuable suggestion.
We have carefully reviewed the formatting of all tables in the manuscript and have made the necessary adjustments to ensure a consistent style throughout. The tables now follow a uniform formatting style, in line with the journal's guidelines. We appreciate your attention to detail and believe these changes improve the clarity and presentation of the manuscript.

Suggestion 5:
Results section needs major revision which demonstrates the training as well the testing results. In the meantime the impacts of each consider parameter in a detailed manner which improves the validity of the approach and quality of the manuscript.

Response:

Dear Reviewer,
Thank you for your valuable comments regarding our manuscript. We fully agree with your suggestion that the Results section needs major revision to demonstrate both training and testing results.
Regarding the training and testing data, we need to candidly explain that due to technical issues (system reinstallation), some of our original training data and curves were not properly preserved. To ensure the reliability and completeness of our research, we have taken the following remedial actions:

1. Re-conducted part of the experimental data collection
2. Fully documented the new training and testing processes
3. Supplemented detailed comparative experimental data in sections 4.3.1 and 4.3.2 of the manuscript
4. Added comprehensive analysis of both training and testing curves

Through these revisions, we have:

- More systematically demonstrated the performance of our proposed method
- Strengthened the reliability of our experimental results
- Provided in-depth analysis of how each parameter impacts system performance

These modifications have not only enhanced the academic quality of our manuscript but have also provided us with valuable research experience. We sincerely appreciate your constructive feedback, which has played a crucial role in improving the quality of our paper. All changes in the revised manuscript have been highlighted in yellow for your convenience.
We are more than willing to make further improvements if you have any additional suggestions or concerns.

Best regards
