# Supplementary material for: IDNet: An inception-like deformable non-local network for projection compensation over non-flat textured surfaces
Source: PLoS One. 2025 May 20;20(5):e0318812. doi: 10.1371/journal.pone.0318812 (PMC12091798; doi:10.1371/journal.pone.0318812)
Supplement: Attachment — Submitted filename: review.docx [file pone.0318812.s001.docx]

The manuscript proposes a novel deep learning method aimed at projection compensation over non-flat textured surfaces, achieving progress in both geometric and photometric compensation. However, there are several aspects that require improvement, and I recommend a major revision. The following are my suggestions:

1. The photometric compensation in this study mainly relies on multi-scale feature extraction and global attention mechanisms, lacking explicit modeling of lighting conditions and surface normal relationships. I recommend that the authors consider incorporating surface normal information into the photometric compensation process, which could allow for a more accurate adjustment of lighting effects and further improve the quality of the compensation results. Integrating normal information can better address the needs of projection compensation under non-uniform lighting, resulting in more realistic outputs.
2. The current experimental results lack detailed quantitative analysis for different types of surfaces (e.g., highly reflective or dark-textured surfaces). I suggest adding performance comparisons under various surface conditions in the experimental section to comprehensively demonstrate IDNet’s adaptability and robustness across diverse scenarios. This would help strengthen the argument for the method and showcase its effectiveness in practical applications.
3. I suggest further clarification in the methodology section regarding the roles and interactions of different modules in IDNet, such as how the multi-scale feature extraction collaborates with deformable convolutions. Providing a detailed explanation of the contributions of these modules in handling geometric and photometric distortions would help readers better understand the innovations of the method. This will emphasize the core advantages of IDNet in compensation tasks and help readers clearly grasp the design motivations behind each component.
4. While IDNet uses deformable convolutions and non-local attention mechanisms to capture surface geometric information, its ability to compensate for complex non-flat surfaces, such as those with significant bumps or fine textures, may still be limited. I recommend considering how to better incorporate surface normal information into the method to improve the accuracy of geometric compensation. Photometric stereo techniques have proven effective in recovering high-precision shapes and normal information, especially for complex surfaces. I suggest referencing these works to gain insights into how to capture and utilize geometric details more precisely in the compensation process.

[1] Woodham R J. Photometric method for determining surface orientation from multiple images[J]. Optical engineering, 1980, 19(1): 139-144.

[2] Shi B, Mo Z, Wu Z, et al. A Benchmark Dataset and Evaluation for Non-Lambertian and Uncalibrated Photometric Stereo[J]. IEEE Transactions on Pattern Analysis and Machine Intelligence, 2019, 41(02): 271-284.

[3] Ju Y, Lam K M, Xie W, et al. Deep Learning Methods for Calibrated Photometric Stereo and Beyond[J]. IEEE Transactions on Pattern Analysis and Machine Intelligence, 2024.
