# Supplementary material for: IDNet: An inception-like deformable non-local network for projection compensation over non-flat textured surfaces
Source: PLoS One. 2025 May 20;20(5):e0318812. doi: 10.1371/journal.pone.0318812 (PMC12091798; doi:10.1371/journal.pone.0318812)
Supplement: Attachment — Submitted filename: Response to Academic Editor.docx [file pone.0318812.s002.docx]

**Journal requirements:**

1. Please ensure that your manuscript meets PLOS ONE's style requirements, including those for file naming. The PLOS ONE style templates can be found at

<https://journals.plos.org/plosone/s/file?id=wjVg/PLOSOne_formatting_sample_main_body.pdf>

and

<https://journals.plos.org/plosone/s/file?id=ba62/PLOSOne_formatting_sample_title_authors_affiliations.pdf>

**Response:**

Dear Editor,

Thank you for your guidance regarding the formatting requirements for PLOS ONE. We have carefully revised our manuscript to adhere to PLOS ONE’s style guidelines, including formatting the main body, title page, authorship, and affiliations according to the provided templates. Additionally, we have ensured that all files are appropriately named as per the journal’s requirements.

We hope the revised submission meets the necessary standards, and we are happy to make further adjustments if needed. Thank you for your time and assistance.

2. Please note that PLOS ONE has specific guidelines on code sharing for submissions in which author-generated code underpins the findings in the manuscript. In these cases, all author-generated code must be made available without restrictions upon publication of the work. Please review our guidelines at https://journals.plos.org/plosone/s/materials-and-software-sharing#loc-sharing-code and ensure that your code is shared in a way that follows best practice and facilitates reproducibility and reuse.

**Response:**

Thank you for your reminder regarding the code-sharing guidelines for PLOS ONE submissions. We are pleased to inform you that the code underpinning the findings in our manuscript will be made openly available on GitHub at the following address: <https://github.com/ZYQ121/IDNet>.

3. Thank you for stating the following financial disclosure:

“This research was funded by Jilin Province Science and Technology Development Plan Project, grant number: (20230101179JC) National Natural Science Foundation of China Youth Fund Project, grant number: (No. 61702051), Jilin Province Science and Technology Development Plan Project, grant number: (20200403188SF).”

Please state what role the funders took in the study.  If the funders had no role, please state: "The funders had no role in study design, data collection and analysis, decision to publish, or preparation of the manuscript."

If this statement is not correct you must amend it as needed.

Please include this amended Role of Funder statement in your cover letter; we will change the online submission form on your behalf.

**Response:**

Thank you for your guidance regarding the financial disclosure. We have updated the statement to include the role of the funders in the study. The amended statement is as follows:

"This research was funded by Jilin Province Science and Technology Development Plan Project, grant number: (20230101179JC), National Natural Science Foundation of China Youth Fund Project, grant number: (No. 61702051), and Jilin Province Science and Technology Development Plan Project, grant number: (20200403188SF). The funders had no role in study design, data collection and analysis, decision to publish, or preparation of the manuscript."

4. Thank you for stating the following in the Acknowledgments Section of your manuscript:

“This research was funded by Jilin Province Science and Technology Development Plan Project, grant number: (20230101179JC) National Natural Science Foundation of China Youth Fund Project, grant number: (No. 61702051), Jilin Province Science and Technology Development Plan Project, grant number: (20200403188SF).”

We note that you have provided funding information that is currently declared in your Funding Statement. However, funding information should not appear in the Acknowledgments section or other areas of your manuscript. We will only publish funding information present in the Funding Statement section of the online submission form.

Please remove any funding-related text from the manuscript and let us know how you would like to update your Funding Statement. Currently, your Funding Statement reads as follows:

“This research was funded by Jilin Province Science and Technology Development Plan Project, grant number: (20230101179JC) National Natural Science Foundation of China Youth Fund Project, grant number: (No. 61702051), Jilin Province Science and Technology Development Plan Project, grant number: (20200403188SF).”

Please include your amended statements within your cover letter; we will change the online submission form on your behalf.

**Response:**

Thank you for your helpful comment regarding the placement of funding information in our manuscript. We apologize for the oversight in including funding details in the Acknowledgments section of our manuscript.

We have also included this amended Funding Statement in the cover letter, and we appreciate your assistance in updating the online submission form on our behalf.

Thank you again for your attention to this matter. Please let us know if there are any further changes needed.

5. We note that your Data Availability Statement is currently as follows: [All relevant data are within the manuscript and its Supporting Information files.]

Please confirm at this time whether or not your submission contains all raw data required to replicate the results of your study. Authors must share the “minimal data set” for their submission. PLOS defines the minimal data set to consist of the data required to replicate all study findings reported in the article, as well as related metadata and methods (https://journals.plos.org/plosone/s/data-availability#loc-minimal-data-set-definition).

For example, authors should submit the following data:

- The values behind the means, standard deviations and other measures reported;

- The values used to build graphs;

- The points extracted from images for analysis.

Authors do not need to submit their entire data set if only a portion of the data was used in the reported study.

If your submission does not contain these data, please either upload them as Supporting Information files or deposit them to a stable, public repository and provide us with the relevant URLs, DOIs, or accession numbers. For a list of recommended repositories, please see https://journals.plos.org/plosone/s/recommended-repositories.

If there are ethical or legal restrictions on sharing a de-identified data set, please explain them in detail (e.g., data contain potentially sensitive information, data are owned by a third-party organization, etc.) and who has imposed them (e.g., an ethics committee). Please also provide contact information for a data access committee, ethics committee, or other institutional body to which data requests may be sent. If data are owned by a third party, please indicate how others may request data access.

**Response:**

Thank you for your comment regarding the Data Availability Statement. We would like to confirm that our submission includes all necessary data to replicate the study's findings. The relevant raw data, including the values behind the means and the minimal local experimental dataset, are provided within the manuscript and its supplementary materials. The proposed dataset and supplementary materials have been uploaded to the following website:

<https://datadryad.org/stash/share/3cx6cQN8lhgLOUnt4_N5HrLmijOaImEV0fngxCFNya4>

We appreciate your guidance, and please do not hesitate to let us know if any further information or adjustments are required.

6. PLOS requires an ORCID iD for the corresponding author in Editorial Manager on papers submitted after December 6th, 2016. Please ensure that you have an ORCID iD and that it is validated in Editorial Manager. To do this, go to ‘Update my Information’ (in the upper left-hand corner of the main menu), and click on the Fetch/Validate link next to the ORCID field. This will take you to the ORCID site and allow you to create a new iD or authenticate a pre-existing iD in Editorial Manager.

Thank you for your reminder regarding the ORCID iD requirement. I have confirmed that my ORCID iD is valid and updated in Editorial Manager. I have followed the instructions to authenticate my ORCID iD, and all necessary information is now correctly recorded.

Thank you again for your assistance.

Note: We apologize for an error in the skip connection in Figure 1 of our initial manuscript submission. This inaccuracy has been corrected in the revised version.

Best regards,
